# Integrating Vaginal Microbiome Test Results into Shared Decision Making during In Vitro Fertilization Care

**DOI:** 10.1177/0272989X261463217

**Published:** 2026-07-15

**Authors:** Rivka Koedooder, Xu Shan Gao, Sam Schoenmakers, Andries E. Budding, Jesper M. J. Smeenk, Jonathan D. de Jonge, Joop S. E. Laven

**Affiliations:** 1 Division of Reproductive Endocrinology and Infertility, Department of Obstetrics and Gynaecology, Erasmus University Medical Center, Rotterdam, The Netherlands; 2 Division Obstetrics, Department of Obstetrics and Gynaecology, Erasmus University Medical Center, The Netherlands; 3 inBiome B.V., Amsterdam, The Netherlands; 4 Department of Reproductive Medicine, Elisabeth-TweeSteden Hospital, Tilburg, the Netherlands; 5 ARTPred B.V., Oude Meer, The Netherlands

**Keywords:** Vaginal microbiome, in vitro fertilization (IVF), shared decision making, prognostic testing, treatment postponement

## Abstract

**Background:**

The vaginal microbiota test predicts the success of in vitro fertilization (IVF), but with no therapy available to improve a low profile, couples must decide whether to proceed or postpone treatment. We aim to examine how couples interpret vaginal microbiome results and make postponement decisions within a shared decision making (SDM) framework.

**Methods:**

Women undergoing IVF or IVF–intracytoplasmic sperm injection (IVF-ICSI) treatment at 2 Dutch hospitals received the ReceptIVFity test™, which classified the vaginal microbiome as high (52.6% chance of conception), medium (23.6%), or low (5.9%) profile based on predicted implantation success after a fresh embryo transfer. Physicians discussed the results with couples using SDM, after which the couples decided whether to proceed or postpone treatment. The primary outcome was the patients’ perceived involvement in shared decision making, assessed with the SDM-Q-9 questionnaire. The secondary outcome was the proportion of couples postponing treatment after a low microbiome profile.

**Results:**

Between October 2018 and November 2020, 728 women were enrolled. SDM-Q-9 responses showed high perceived involvement overall but lower scores for “exploring options,” reflecting limited alternatives when the choice is to proceed or postpone treatment. A low profile was found in 35.4% (258/728). After the SDM consultation, 49.6% (128/258) chose to postpone treatment, with postponement rates increasing to over 80% among couples in later IVF cycles. Decisions were influenced by personal, emotional, and practical considerations, including the Dutch insurance reimbursement system (3 insured IVF or IVF-ICSI cycles regardless of postponement) and the absence of effective treatment to modify a low profile.

**Conclusions:**

These findings demonstrate that couples can understand and use prognostic information when supported by SDM and that the ReceptIVFity test™ facilitated discussion about chances of success, timing of treatment, decisions to proceed or postpone, and personal values.

**Highlights**

- Couples can understand and use prognostic information when supported by shared decision making, and the vaginal microbiome test facilitated discussions about chances of success, treatment timing, and personal values in decision making.
- Nearly half of couples with a low predicted chance of success chose to postpone treatment after discussing the results within a shared decision making consultation.
- Decision making was influenced not only by prognostic information but also by previous IVF cycles and contextual factors such as insurance coverage.

## Introduction

In vitro fertilization (IVF) and intracytoplasmic sperm injection (IVF-ICSI) represent major advances in reproductive medicine, offering millions of couples worldwide the possibility of conceiving a child. Yet, despite ongoing scientific refinement and technological sophistication, the probability of achieving a pregnancy per treatment cycle remains limited, typically between 20% and 25%.^1^ Each failed cycle can impose considerable physical, emotional, and financial strain.^2,3^ Repeated unsuccessful attempts can lead to distress, loss of control, and uncertainty about whether to continue, pause, or stop treatment altogether. Communicating with couples at these moments requires both clinical clarity and emotional sensitivity to address the uncertainty and vulnerability that accompany fertility treatment.

One emerging factor associated with the success of IVF is the vaginal microbiota, the community of microorganisms that inhabit the vagina.^4,5^ A *Lactobacillus*-dominant vaginal microbiome has long been considered characteristic of a healthy vaginal environment, as lactobacilli inhibit the growth of other bacteria and pathogens by producing lactic acid.^6,7^ The absence of lactobacilli or the presence of bacteria such as *Gardnerella vaginalis* and *Escherichia coli* have been linked to adverse IVF or IVF-ICSI outcomes.^8–11^ Because the vaginal microbiota can fluctuate over time, its stability and composition have become important in reproductive research. Longitudinal studies have shown that spontaneous transitions toward a more favorable microbiota can occur within weeks, even without medical intervention.^12–14^ Communicating this probabilistic and dynamic information requires careful balancing of medical evidence with patient values to support informed decision making in fertility care.

The CE-certified ReceptIVFity test™ was developed to provide couples with a predictive vaginal microbiota profile before starting IVF or IVF-ICSI.^8,15,16^ These profiles are associated with the following probabilities of achieving a pregnancy after a fresh embryo transfer: 52.6% for a high profile, 23.6% for a medium profile, and only 5.9% for a low profile.^8^ The 3 profiles reflect the degree of *Lactobacillus* dominance in the vaginal microbiota as measured by the IS-Pro technique.^8^ A high profile indicates a *Lactobacillus*-dominant microbiome, a medium profile reflects partial *Lactobacillus* presence, whereas a low profile is more diverse and defined by criteria including a relative *Lactobacillus* load <20%, a relative load of *Lactobacillus jensenii* >35%, the presence of *Gardnerella vaginalis IST1*, or proteobacteria >28% of the total bacterial load.^8^ While this prognostic information may help couples better understand their likelihood of treatment success, it also introduces uncertainty. A low ReceptIVFity profile indicates a low probability of success, with currently no therapeutic intervention available to improve an unfavorable microbiome. Couples must therefore decide, often under emotional and time pressure, whether to proceed with low odds or postpone treatment in hopes of spontaneous improvement. Shared decision making (SDM) is expected to facilitate such decisions by aligning medical evidence with patient preferences.^17,18^ This study applies a 3-step SDM model consisting of 1) introducing choice, in which couples were informed that the test result could influence treatment timing while emphasizing that the decision to proceed or postpone remained their own; 2) describing options, in which the advantages and disadvantages of continuing or temporarily postponing treatment were discussed; and 3) exploring preferences, in which couples were encouraged to reflect on personal, emotional, and contextual factors before making a decision.^19^ However, there is limited empirical evidence on how couples interpret and act upon prognostic information within this type of SDM process in fertility care, particularly when the clinical options are limited to proceed now or postpone treatment. This approach was used to examine how couples responded to such prognostic information when using the test for the first time.

This study therefore explores how couples undergoing IVF or IVF-ICSI interpret and act upon their vaginal microbiome results within an SDM process. Specifically, we examine 1) how prognostic information is communicated and understood by couples and 2) how these couples justify their decisions to postpone or proceed with treatment, revealing the communicative challenges of SDM. By focusing on the communication process, this study contributes to a broader understanding of how SDM can support patient autonomy and informed decision making under prognostic uncertainty in fertility care.

## Materials and Methods

### Study Design and Setting

This was a prospective cohort study conducted between October 2018 and November 2020 at 2 fertility centers in the Netherlands: Erasmus University Medical Centre (tertiary care) and Elisabeth-TweeSteden Hospital (secondary care). All participating women received the ReceptIVFity test™ prior to initiating their first, second, or third IVF or IVF-ICSI cycle. All women were given the choice to undergo a routine IVF or IVF-ICSI procedure irrespective of their test result. Patients had the option to repeat the ReceptIVFity™ test following treatment discontinuation based on the test result. Similarly, in case the previous cycle did not lead to a successful pregnancy, patients could repeat the test.

The study did not include a control group, as withholding the test was ethically and practically infeasible. This observational design was therefore appropriate to investigate decision making in a real-world setting in which all patients were offered SDM consultations.

### Study Population

Eligible participants were women scheduled for an IVF or IVF-ICSI treatment cycle at 2 large hospitals in the Netherlands, recruited between October 2018 and November 2020. Women visited the outpatient clinic of Reproductive Endocrinology & Infertility for an intake for an IVF or IVF-ICSI treatment, during which they also received information about the study. The fertility specialist or research physician provided an information letter and patient information leaflet and ensured that participants had reviewed all information. Written informed consent was obtained prior to enrollment. Women starting a subsequent IVF cycle were contacted by telephone by the fertility specialist. Women whose test could not be analyzed due to preanalytical errors were excluded. In the Netherlands, health care is organized through mandatory basic health insurance that covers essential medical care for all residents. This standard package includes up to 3 reimbursed IVF or IVF-ICSI cycles for women aged 42 y or younger. To ensure that decisions reflected medical and personal preferences rather than financial constraints, only women whose IVF treatment was reimbursed by their health insurance were included.

### The ReceptIVFity Test

The ReceptIVFity test™ involves self-collection of a vaginal swab prior to the start of the hormonal treatment for IVF or IVF-ICSI. To avoid biased test results, the test was not performed during periods of menstrual flow or irregular blood loss.^20,21^ The vaginal swab was subsequently analyzed by the IS-Pro technique, a validated eubacterial molecular technique that provides results within 1 d.^22^ The vaginal bacterial composition was analyzed and subsequently categorized into low, medium, or high profiles, corresponding with the predicted chance of conceiving following a fresh embryo transfer of approximately 5.9%, 23.6%, and 52.6%, respectively. The ReceptIVFity™ test was provided without cost to patients in this study.

### Shared Decision Making

SDM is an approach that takes into account both the clinical evidence for treatment efficacy as well as the couple’s preferences.^23^ The SDM model we used is based on 3 key steps.^19^ First, introducing choice: physicians informed couples that the test results could influence the timing of treatment but that starting or postponing remained their own decision. Second, describing options: based on the test result, physicians discussed the advantages and disadvantages of either proceeding with or temporarily postponing treatment. Patients received a visual decision aid (SDM cards) outlining these options (Supplementary Figure 1). Finally, the last step was exploring preferences: couples were encouraged to reflect on personal, emotional, and contextual factors before deciding. In case the patient decided to postpone the treatment, the test was repeated.

Prior to study initiation, all health care professionals involved in the study (research nurses, gynecologists, and fertility physicians) received standardized training. This training, conducted shortly before study initiation, consisted of a 2-h workshop covering background information of the ReceptIVFity™ test, logistics from patient inclusion to communication of the test results, introducing a 3-step model of SDM (choice, option, and decision talk),^19^ and the use of the SDM cards in clinical practice (Supplementary Figure 1). This approach aimed to ensure that each patient received sufficient information to support SDM.

### SDM Questionnaire (SDM-Q-9)

All women were asked to complete the Shared Decision-Making Questionnaire (SDM-Q-9) immediately after the test results were discussed and a decision regarding treatment was made (Supplementary Figure 2). The SDM-Q-9 is a validated instrument measuring the extent to which patients perceive involvement in SDM across 9 items scored from 0 (*completely disagree*) to 5 (*completely agree*), yielding a total score from 0 to 45.^24^ This questionnaire was validated for the Dutch language.^25^ The primary analysis used the total sum score to compare overall SDM across microbiome profiles. Item-level analyses were used to examine specific aspects of the decision-making process.

### Outcome Measures

The primary outcome was patients’ perception of the shared decision making process, measured with the SDM-Q-9 questionnaire. The secondary outcome was the proportion of couples who chose to postpone IVF or IVF-ICSI treatment following a low microbiome profile and SDM consultation. Other outcomes involved exploring demographic and clinic factors associated with decision making, such as age, ethnicity, education level, and reproductive history.

### Statistical Analyses

Continuous variables (eg, age, body mass index [BMI], and months trying to conceive) were examined for distribution and are presented as mean (SD) or median (Q1–Q3), as appropriate. Baseline characteristics between SDM-Q-9 responders and nonresponders were compared using chi-square tests for categorical variables, independent-samples *t* tests for normally distributed continuous variables, and Mann–Whitney *U* tests for nonnormally distributed continuous variables.

SDM-Q-9 item scores, measured on a 6-point Likert scale and recoded to a 0 to 5 scale, were analyzed at both the item level and as a total sum score (range 0–45). Differences in individual SDM-Q-9 item scores across low, medium, and high microbiome profile groups were assessed using Kruskal–Wallis tests. The overall SDM-Q-9 sum score was also compared between profile groups using the Kruskal–Wallis test.

Postponement rates following a low microbiome profile were reported descriptively as proportions, stratified by profile and treatment cycle. Categorical variables, including ethnicity, education level, causes of subfertility, and ReceptIVFity test™-result profiles, were summarized as counts and percentages. Univariate associations between these variables and the postponement decision among women with a low profile were evaluated using chi-square or Fisher’s exact tests for categorical variables and Mann–Whitney *U* tests for continuous variables. All analyses were performed in R version 4.3.2 (ucrt, October 31, 2023; R Foundation for Statistical Computing, Vienna, Austria). Two-sided *P* values <0.05 were considered statistically significant.

## Results

### Baseline Characteristics

A total of 748 women performed the ReceptIVFity™ test for the first time prior to the start of their ovarian stimulation for IVF or IVF-ICSI, of whom 20 participants were excluded. The remaining 728 women were available for the current analysis. Of these 728 women, 150 women underwent a second test, 26 a third test, and 3 patients a fourth test (Figure 1). The main reasons for repetitive testing were a recurrent low profile or repetition following an unsuccessful IVF or IVF-ICSI cycle.

**Figure 1.**
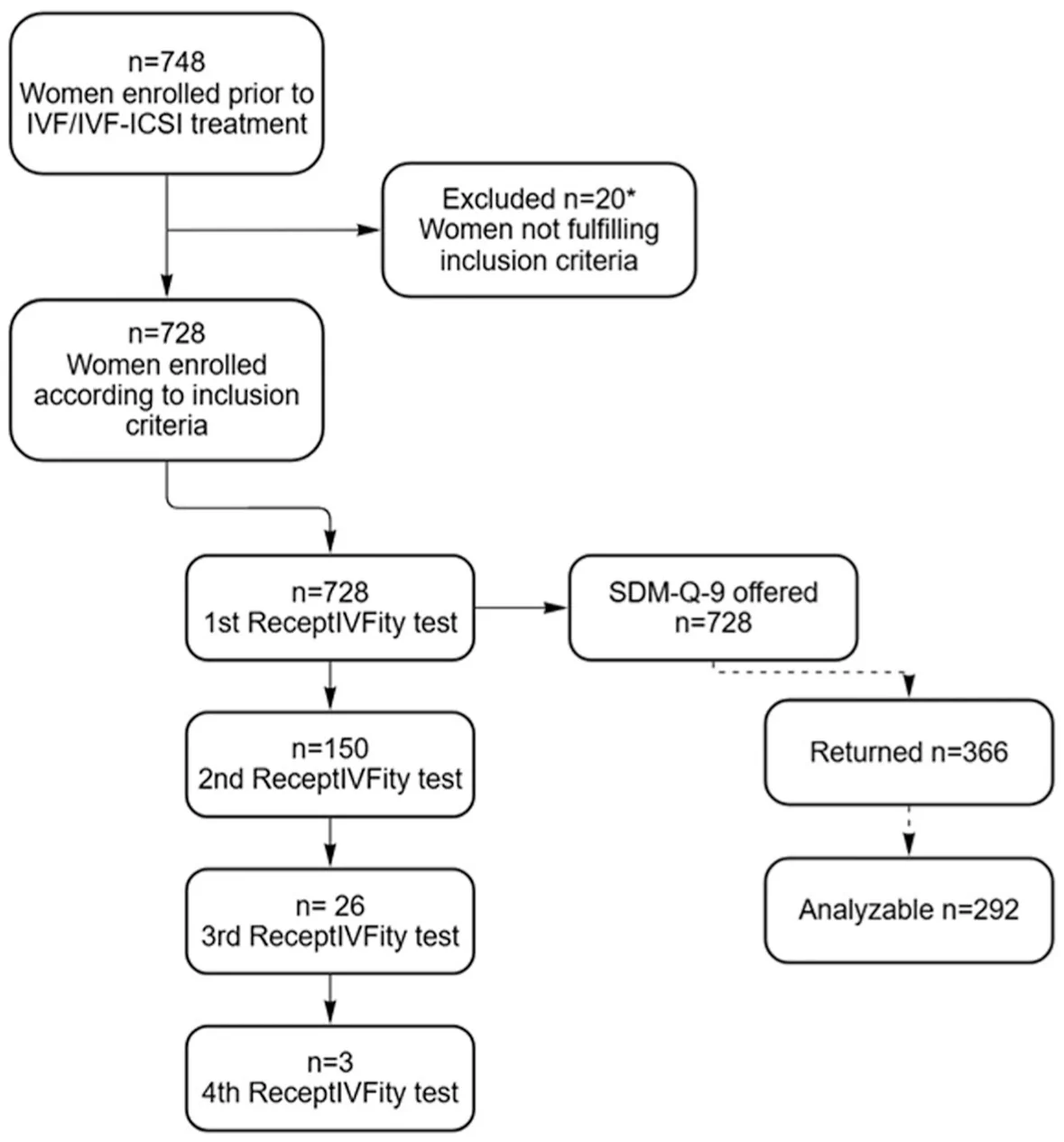
Flowchart of study population. This flowchart illustrates the selection process of the study population for the current analysis. A total of 748 women performed the ReceptIVFity test™ for the first time prior to starting ovarian stimulation for in vitro fertilization (IVF) or IVF–intracytoplasmic sperm injection (IVF-ICSI). Twenty participants were excluded, leaving 728 women available for analysis. Among these, 150 women underwent a second test, 26 underwent a third test, and 3 underwent a fourth test. *Reasons for exclusion (*n* = 20): already pregnant before the test results were discussed (*n* = 2), women were about to start their fourth or fifth IVF or IVF-ICSI treatment cycle (*n* = 10), women underwent an embryo transfer of a cryopreserved embryo (*n* = 4), test results did not meet minimal quality laboratory requirements due to insufficient bacterial load (*n* = 2), and tests never arrived at the laboratory (*n* = 2). The SDM-Q-9 was offered to all 728 women, but only 366 (50%) returned a questionnaire and 292 (80%) were analyzable SDM-Q-9 responses after exclusions.

Approximately 84% of the women (*n* = 609) were tested before their first assisted reproductive technology cycle, and male factor subfertility was the most common cause of subfertility (44.0%). A low profile was observed in 35.4% (258/728) of the women, a medium profile in 14.8% (108/728), and a high profile in 49.7% (362/728) (Table 1).

**Table 1.** Baseline Characteristics.^a^

	Total *N* = 728
Demographic characteristics	
Age, y	34.1 (4.7)
Body mass index, kg/m^2^	24.6 (4.3)
Ethnicity	
Northwestern European	495 (68.0)
Mediterranean	71 (9.8)
Asian	48 (6.6)
Hindu	25 (3.4)
African	44 (6.0)
Unknown/other	45 (6.2)
Education level	
Elementary/secondary education	36 (4.9)
Lower vocational education	294 (40.4)
Higher vocational or university	385 (52.9)
Unknown	33 (4.5)
Reproductive health characteristics	
Months trying to conceive	32.0 (19-49)
Primary subfertility	
Yes	387 (53.2)
No	341 (46.8)
Causes of subfertility	
Male factor subfertility	320 (44.0)
Ovulation disorder	50 (6.9)
Fallopian tube blockage	44 (6.0)
Unexplained	124 (17.0)
Combination	157 (21.6)
Unknown/other	33 (4.5)
Results of ReceptIVFity test	
Low	258 (35.4)
Medium	108 (14.8)
High	362 (49.7)
IVF or IVF-ICSI cycle	
First	609 (83.7)
Second	77 (10.6)
Third	42 (5.8)

ICSI, intracytoplasmic sperm injection; IVF, in vitro fertilization.

a This table presents the demographic and reproductive health characteristics of the 728 women included in the study. Most women (83.7%) underwent testing before their first IVF or IVF-ICSI cycle, with male factor subfertility being the primary cause (44.0%). Values are given as mean ± SD, number (%), and median (interquartile range).

### SDM-Q-9 Outcomes

#### Patient perceptions of SDM

A total of 366 SDM-Q-9 were returned after the consultation (366/728, 50%). Nineteen questionnaires were incomplete, and 26 could not be assigned to a test result group. Furthermore, 3 questionnaires related to a cryopreserved cycles, 2 lacked information on whether treatment was continued or postponed, and 24 were repeat questionnaires following a previous test. The remaining 292 questionnaires (80%) were included in the analysis. These were distributed across the low (*n* = 117, 40%), medium (*n* = 41, 14%), and high (*n* = 134, 46%) profile groups.

The distribution of ReceptIVFity test™ result profiles differed significantly between responders and nonresponders (*P* = 0.011), with a higher proportion of high-profile patients among nonresponders (54%) and a higher proportion of low-profile patients among responders (40%). No significant differences were found in age, BMI, ethnicity, education level, causes of subfertility, or months trying to conceive (Supplementary Table 1).

Overall SDM-Q-9 sum scores were high across all profiles, with median scores of 38 (Q1–Q3 32–43) in the low group, 40 (33–45) in the medium group, and 36 (29–43) in the high group. There were no statistically significant differences between profiles (*P* = 0.140).

At the item level, median scores were consistently high across all 9 questions, predominantly ranging between 4 and 5, indicating a clear ceiling effect. Medians and interquartile ranges largely overlapped between profiles, reflecting minimal variability in perceived SDM. No statistically significant differences were observed for 8 items. While statistical significance was observed for question 9 (*P* = 0.048), this was not clinically meaningful, as median scores were identical across profiles, and responses were predominantly concentrated at the upper end of the scale.

Furthermore, item-level analyses revealed communication insights. Among women with a low profile, 97.5% felt the doctor helped them to understand all information (Q5), and 97.4% felt an agreement was reached on how to proceed (Q9). These responses indicate that most women felt adequately informed and supported in the decision-making process. However, 23.1% reported low scores (≤2) for the item “My doctor told me that there are different options for treating my condition (Q3),” and 20.6% for “My doctor and I weighed the different treatment options thoroughly (Q7)” (Supplementary Table 2). These lower ratings likely reflect the structural limitations of SDM in a prognostic context without therapeutic alternatives, where patients may perceive few meaningful options.

### Communicating Prognostic Information and Initial Decisions

The second research question explored how couples used prognostic information within SDM to decide whether to proceed or postpone treatment.

After receiving the test results and participating in SDM, 49.6% (128/258) of couples with a low profile chose to temporarily postpone treatment. In contrast, only 6.5% (7/108) with a medium profile and 3.9% (14/362) with a high profile did. The likelihood of postponement among low-profile couples increased substantially with each treatment cycle: 43.5% before the first cycle, 77.8% before the second, and 82.4% before the third. Similar patterns were observed for medium profiles (Table 2). These patterns show that the prognostic information delivered via SDM influenced how couples understood their reproductive chances and informed their choices about treatment timing, further influenced by the number of previous cycles.

**Table 2.** Decisions Based on the First ReceptIVFity Test Result.^a^

	Temporarily Postponing Treatment	Continuing Treatment
First cycle (*n* = 609)		
Low (*n* = 214)	93 (43.5)	121 (56.5)
Medium (*n* = 92)	4 (4.3)	88 (95.7)
High (*n* = 303)	11 (3.6)	292 (96.4)
Second cycle (*n* = 77)		
Low (*n* = 27)	21 (77.8)	6 (22.2)
Medium (*n* = 10)	1 (10.0)	9 (90.0)
High (*n* = 40)	1 (2.5)	39 (97.5)
Third cycle (*n* = 42)		
Low (*n* = 17)	14 (82.4)	3 (17.6)
Medium (*n* = 6)	2 (33.3)	4 (66.7)
High (*n* = 19)	2 (10.5)	17 (89.5)

a This table shows the decisions of couples to postpone treatment after the first ReceptIVFity test, stratified by treatment cycle. The percentage of couples postponing treatment increased with each additional in vitro fertilization/in vitro fertilization–intracytoplasmic sperm injection cycle: 43.5% in the first cycle, 77.8% in the second, and 82.4% in the third cycle. Similar trends were observed for couples with a medium profile. Values are given as number (%).

### Factors Influencing Decisions to Postpone or Continue Treatment

To explore factors associated with decision making after a low profile, univariate analyses were performed between couples who postponed treatment (*n* = 128) and those who continued (*n* = 130). No significant differences were observed in age, causes of subfertility, months trying to conceive, or reproductive history. Differences were observed for ethnicity (*P* = 0.009), BMI (*P* = 0.042), and education level (*P* = 0.015). The postponement group included a higher proportion of women with a Northwestern European background (73% vs 62%) and a higher proportion with higher vocational or university education (46% vs 31%). Mean BMI was 25.3 (SD 4.8) among women who postponed and 24.2 (SD 4.3) among those who continued.

Reasons for continuing treatment despite a low result were documented in the clinical notes for approximately 40% of the couples (53/130). Several couples reported multiple reasons. Documented reasons could be divided into 3 main categories: 1) medical considerations, 2) practical planning, and 3) personal or emotional reasons. Medical reasons included incipient ovarian failure (*n* = 2), low anti-Müllerian hormone (*n* = 4), advanced maternal age of 42 y (*n* = 10), or the belief that subfertility was due primarily to male factors (*n* = 3). Practical considerations included a long waiting period before IVF initiation (*n* = 11) and work or holiday planning (*n* = 5). Personal or emotional reasons included a predetermined decision to start regardless of the test result (*n* = 8), previous success after IVF (*n* = 2), the belief that postponement offered no benefit given the lack of therapeutic intervention (*n* = 3), and acute personal or emotional circumstances (n = 5). For the remaining couples, no specific reason beyond their wish to proceed was recorded.

Despite being informed by prognostic information, couples’ final decisions consistently integrated emotional, practical, and experiential factors, illustrating the multidimensional nature of SDM in fertility care.

### Postponement Decisions in Medium/High Profiles

Some couples with medium (*n* = 7) or high (*n* = 14) profiles also postponed or discontinued treatment for reasons unrelated to their microbiome result. Couples with a medium profile reported factors such as travel to a Zika-endemic area (*n* = 2), pending fertility-related medical procedures (*n* = 2; clinical genetics, hysteroscopy), nonmedical reasons (*n* = 1), and unspecified (*n* = 2).

Couples with a high profile reported discontinuation due to medical reasons (*n* = 2; treatment of viral hepatitis, revalidation after spinal injury), pending fertility-related medical procedures (*n* = 4; hysteroscopy, laparoscopy, uterine fibroid resection, microsurgical epididymal sperm aspiration), lifestyle changes (*n* = 4; weight loss, fitness), or personal circumstances (*n* = 3; relationship or unspecified); for one couple no reason was given. These findings indicate that SDM interactions frequently extended beyond the test outcome to include discussion of personal and contextual factors shaping couples’ readiness for treatment.

### Repetition of the ReceptIVFity Test

Couples who postponed treatment after a low profile were offered a repeat test after approximately 3 mo. Of the 128 women who postponed after the first test, 68.0% (n = 87) underwent a second test. Among women with a persistently low profile, 35.6% (21/59) chose to postpone again at the second test (approximately 3 mo after the first) and 41.7% (5/12) at the third test (approximately 6 mo after the first); none of the 2 women with a low profile at the fourth test postponed. Repeated test results were discussed using SDM, and decisions about postponement were revisited over time as new prognostic information became available.

## Discussion and Conclusion

The current study examined how couples undergoing IVF or IVF-ICSI treatment interpreted and acted upon prognostic information based on their vaginal microbiome (ReceptIVFity test) during SDM consultations.^8^ The findings show that couples were able to understand and use prognostic information to make informed decisions about the timing of their treatment. At the same time, the absence of treatment options can complicate informed decision making, as couples may find it difficult to understand the value of postponement when there are no active treatments available. In addition, the role of the insurance system and the number of remaining cycles influenced couples’ decisions.

### Understanding and Interpreting Prognostic Information

Overall, patients are able to decide whether to continue or postpone their treatment. This study highlights how SDM empowers couples by involving them in decisions about their care and ensuring that their preferences are respected. About 20% to 25% of patients with a low profile gave lower scores on “exploring different options”. This aligns with the SDM theory, which shows that discussion is more limited when postponing treatment is the only alternative in the absence of effective treatment. This reflects an inherent limitation of SDM in prognostic settings rather than a shortcoming of communication. Similar challenges are seen in oncology, where decisions concern when or whether to act rather than choosing between alternative treatments (drug A vs drug B). Our findings highlight that integrating predictive tools such as the ReceptIVFity test into the SDM process remains important even when few treatment options exist, as it helps patients manage uncertainty and stay actively involved in their decisions.

### Decision Making about Postponing Treatment

The second research question examined how couples made decisions to postpone or continue treatment after receiving their vaginal microbiome profile. Almost 50% of the couples with a low profile chose to postpone treatment, and this proportion increased with each previous failed cycle. Couples who decided to continue treatment despite a low profile often cited personal, emotional, or practical reasons, such as long waiting times, work or travel schedules, or the belief that infertility was caused by other medical factors. These findings demonstrate that SDM in fertility care is shaped by a combination of medical information and individual circumstances. Even when provided with prognostic information, patients interpret and act on risk information based on their own experiences, emotions, and expectations.

Decision making in our study was also influenced by factors such as the Dutch insurance system, which covers 3 full IVF or IVF-ICSI cycles as part of the national basic health plan. Because treatment costs are fully reimbursed, couples have no financial reason to postpone. In other health systems, in which patients initially must pay for treatment themselves or if the insurance company reimburses the treatment only in case of a favorable (medium or high) profile, we expect this could lead to different decisions. This is also reflected in the higher percentages of temporarily postponing treatment before the second and third cycles.

Furthermore, the absence of an effective therapy to improve a low microbiota profile likely played a role in decision making. Although spontaneous improvement can occur, the uncertain timing may lead some couples to restart treatment after waiting for several months. At present, no proven therapy exists to actively modify an unfavorable microbiota. A recent randomized, double-blind trial in subfertile women with a low ReceptIVFity profile found that about 40% shifted spontaneously to a more favorable microbiome within a few months, regardless of probiotic or placebo treatment.^13^ This finding supports postponement as a reasonable and informed option rather than a therapeutic intervention, highlighting the need for careful communication so that patients understand waiting is a realistic choice based on prognosis, not as an active treatment.

### Practice Implications

Integrating predictive tools like the ReceptIVFity test ™ within SDM can help clinicians align care with patient values, support informed timing decisions, and reduce unnecessary interventions, ultimately improving the quality and personalization of fertility care. Communicating uncertain outcomes requires balancing realism with empathy, supporting patients in interpreting risk without losing a sense of hope. Future research should explore how the use of the ReceptIVFity test within SDM influences not only patient experience and communication outcomes but also the efficient use of IVF resources, ensuring that treatment is not only initiated when likely to succeed and avoiding unnecessary cycles that could burden patients and the health care system. It would be valuable to examine whether patients who postpone treatment based on a low profile use their reimbursed IVF or IVF-ICSI cycles more efficiently and cost-effectively than those who proceed immediately or do not use the ReceptIVFity test. This information holds significant implications for health insurance companies and IVF centers, particularly concerning whether the use of the test should be established as standard care and whether physicians can use their professional guidance to strictly advise postponement of treatment in case of a low profile.

### Strengths and Limitations

A major strength of this study is that it captures how couples respond the first time they use the ReceptIVFity test in fertility care, providing insight into how patients interpret prognostic information and make choices in whether to start or postpone treatment. The SDM-Q-9 response rate of 50% was acceptable, although we had hoped for a higher rate given that the questionnaire was brief and designed to be completed immediately following the communication of test results. Reminders and telephone follow-up were provided to encourage timely completion. Notably, the group of responders was representative, with no significant differences in age, BMI, ethnicity, education level, causes of subfertility, or months trying to conceive, supporting the validity of the SDM-Q-9 findings.

## Conclusion

This study shows that couples undergoing IVF are able to understand and use prognostic information when applying a structured SDM process. The ReceptIVFity test not only acted as a prognostic tool but also facilitated discussions about chances of success, treatment timing, the option to postpone treatment, and personal values.

## Supplemental Material

sj-docx-1-mdm-10.1177_0272989X261463217 – Supplemental material for Integrating Vaginal Microbiome Test Results into Shared Decision Making during In Vitro Fertilization Care Supplemental material, sj-docx-1-mdm-10.1177_0272989X261463217 for Integrating Vaginal Microbiome Test Results into Shared Decision Making during In Vitro Fertilization Care by Rivka Koedooder, Xu Shan Gao, Sam Schoenmakers, Andries E. Budding, Jesper M. J. Smeenk, Jonathan D. de Jonge and Joop S. E. Laven in Medical Decision Making

## Data Availability

The data that support the findings of this study are available from the corresponding author upon reasonable request.
