# Supplementary material for: Integrating Vaginal Microbiome Test Results into Shared Decision Making during In Vitro Fertilization Care
Source: Med Decis Making. 2026 Jul 15;46(8):1037–45. doi: 10.1177/0272989X261463217 (PMC13714200; doi:10.1177/0272989X261463217)
Supplement: Supplementary file 1 [file sj-docx-1-mdm-10.1177_0272989X261463217.docx]

**Supplementary Figure 1. Shared Decision-Making cards to discuss the results and determine whether to proceed with or postpone the treatment.**

A) **Low profile - shared decision-making**
   - Your microbiome is currently unfavorable for embryo implantation, it is unlikely to conceive with IVF/IVF-ICSI treatment at this moment.
   - With continuation of the IVF/IVF-ICSI treatment, your predicted chance to conceive is 5%. This is much lower than the average of 33%. These percentages are determined for women prior to the first treatment cycle.
   - This chance can be different for each patient due to personal factors or whether it is not the first treatment cyle.

| **Options** | **Continue treatment** | **Postpone treatment** | **Discontinue treatment** |
| --- | --- | --- | --- |
| **Advantages** | Treatment can be started, despite the small chance of success. For example, when urgency is required for medical reasons. | A new test can easily be done after 3 months, with the chance that the profile will improve to high (chance of success 52%) or medium (chance of success 23%). | Depending on personal circumstances.  Treatment burden is prevented. |
| **Disadvantages** | General disadvantages of treatment, such as physical and mental burden and possibly also costs, while the chance of success is very low. | Longer waiting time prior to the start of the treatment.  There might be a risk that the profile will be low again after 3 months. | Low chance to achieve a pregnancy. |

B) **Medium profile - shared decision-making**
   - Your microbiome is currently neutral for embryo implantation.
   - With continuation of the IVF/IVF-ICSI treatment, your predicted chance to conceive is 23%. This is slightly lower than the average of 33%. These percentages are determined for women prior to the first treatment cycle.
   - This chance can be different for each patient due to personal factors or whether it is not the first treatment cyle.

| **Options** | **Continue treatment** | **Postpone treatment** | **Discontinue treatment** |
| --- | --- | --- | --- |
| **Advantages** | Reasonable chance to conceive with the treatment.  No longer waiting time prior to the start of the treatment.  No risk of a low profile when repeating the test after 3 months. | A new test can easily be done after 3 months, with the chance that the profile will improve to high (chance of success 52%). | Depending on personal circumstances.  Treatment burden is prevented. |
| **Disadvantages** | General disadvantages of treatment, such as physical and mental burden and possibly also costs, while the chance of success is not optimal. | Longer waiting time prior to the start of the treatment.  There might be a risk that the profile will be medium again or even low after 3 months. | Low chance to achieve a pregnancy. |

C) **High profile - shared decision-making**
   - Your microbiome is currently favorable for embryo implantation. However, this is not a guarantee to conceive with the treatment. Severals factors play a role (age, BMI, oocyte/sperm/embryo quality, etc.)
   - With continuation of the IVF/IVF-ICSI treatment, your predicted chance to conceive is 52%. This is much higher than the average of 33%. These percentages are determined for women prior to the first treatment cycle.
   - This chance can be different for each patient due to personal factors or whether it is not the first treatment cyle.

| **Options** | **Continue treatment** | **Postpone treatment** | **Discontinue treatment** |
| --- | --- | --- | --- |
| **Advantages** | Take full advantage of a high microbiome profile. | Depending on personal circumstances. | Depending on personal circumstances.  Treatment burden is prevented. |
| **Disadvantages** | General disadvantages of treatment, such as physical and mental burden and possibly also costs. | Depending on personal circumstances. | Low chance to achieve a pregnancy. |

**Supplementary Figure 2: The 9-item Shared Decision Making Questionnaire (SDM-Q-9)**

**Supplementary Table 1. Baseline characteristics of responders and non-responders of the SDM-Q9**

|  | **Responder s**  **n=292** | **Non-Responders n=436** | ***p*-value** |
| --- | --- | --- | --- |
| **Demographic characteristics** | | | |
| Age | 34.2 (4.8) | 34.2 (4.2) | 0.827a |
| BMI | 24.5 (4.1) | 24.6 (4.4) | 0.617a |
| Ethnicity  Caucasian  Mediterranean  Asian  Hindu  African Unknown/Other | 203 (69.5)  25 (8.6)  19 (6.5)  13 (4.5)  15 (5.1)  17 (5.8) | 292 (67.0)  46 (10.6)  29 (6.7)  12 (2.8)  29 (6.7)  28 (6.4) | 0.682^b^ |
| Education level  Elementary/ Secondary education  Lower vocational education  Higher vocational or university  Unknown | 8 (2.7)  73 (25.0)  124 (42.5)  87 (28.8) | 23 (5.3)  108 (24.8)  184 (42.2)  144(33.0) | 0.278b |
| **Reproductive health characteristics** | | | |
| Months trying to conceive | 32 (18.0-46.2) | 33 (20.0-50.0) | 0.232c |
| Causes of subfertility  Male factor subfertility  Ovulation disorder  Fallopian tube blockage Unexplained  Combination  Unknown/Other | 132 (45.2)  13 (4.5)  15 (5.1)  50 (17.1)  69 (23.6)  13 (4.5) | 188 (43.1)  37 (8.5)  29 (6.7)  74 (17.0)  88 (20.2)  20 (4.6) | 0.311b |
| **Result ReceptIVFity test**  Low  Medium  High | 117 (40.1)  41 (14.0)  134 (45.9) | 136 (31.2)  66 (15.1)  234 (53.7) | 0.011b |

*Baseline demographic and clinical characteristics of study participants stratified by response status (responders, n = 292; non-responders, n = 436). Values are given as mean ± SD years, number (%), and median (IQR). BMI= Body Mass Index (kg/m^2^).aBy One sample t-test, bBy Chi-square test, cBy Mann-Whitney U test*

**Supplementary Table 2. Distribution of SDM-Q9 item scores in the Low profile group**

| **Shared decision-making process steps corresponding to SDM-Q-9 items** | **0** | **1** | **2** | **3** | **4** | **5** |
| --- | --- | --- | --- | --- | --- | --- |
| Q1: My doctor made clear that a decision  needs to be made | 5 (4.3) |  | 3 (2.6) | 7 (5.9) | 29 (24.8) | 73 (62.4) |
| Q2: My doctor wanted to know exactly how I want to be involved in making the decision | 5 (4.3) | 3 (2.6) | 6 (5.1) | 11 (9.4) | 44 (37.6) | 48 (41.0) |
| Q3: My doctor told me that there are different options for treating my condition | 18 (15.4) | 2 (1.7) | 7 (6.0) | 10 (8.5) | 30 (25.6) | 50 (42.7) |
| Q4: My doctor precisely explained the advantages and disadvantages of the treatment options | 8 (6.8) | 2 (1.7) | 1 (0.9) | 11 (9.4) | 29 (24.8) | 66 (56.4) |
| Q5: My doctor helped me understand all the information | 1 (0.9) | 1 (0.9) | 1 (0.9) | 5 (4.3) | 40 (34.2) | 69 (59.0) |
| Q6: My doctor asked me which treatment option I prefer | 9 (7.7) | 1 (0.9) | 5 (4.3) | 12 (10.3) | 41 (35.0) | 49 (41.9) |
| Q7: My doctor and I weighed the different  treatment options thoroughly | 12 (10.3) | 3 (2.6) | 9 (7.7) | 10 (8.5) | 41 (35.0) | 42 (35.9) |
| Q8: My doctor and I selected a treatment  option together | 10 (8.5) | 4 (3.4) | 9 (7.7) | 9 (7.7) | 29 (24.8) | 56 (47.9) |
| Q9: My doctor and I came to an agreement  on how to proceed | 2 (1.7) |  | 1 (0.9) | 10 (8.5) | 27 (23.1) | 77 (65.8) |

*Distribution of responses for each item of the 9-item Shared Decision-Making Questionnaire (SDM-Q9) among patients classified in the Low profile group. For each item, the table shows the number of patients and percentage selecting each SDM-Q9 score (0-5). Scores range from 0 (completely disagree) to 5 (completely agree), with higher scores indicating greater perceived involvement in Shared Decision-making.*
